# Fibroblasts Derived from Human Pluripotent Stem Cells Activate Angiogenic Responses *In Vitro* and *In Vivo*

**DOI:** 10.1371/journal.pone.0083755

**Published:** 2013-12-30

**Authors:** Yulia Shamis, Eduardo A. Silva, Kyle J. Hewitt, Yevgeny Brudno, Shulamit Levenberg, David J. Mooney, Jonathan A. Garlick

**Affiliations:** 1 Program in Cell, Molecular and Developmental Biology, Sackler School of Graduate Biomedical Sciences, Tufts University School of Medicine, Boston, Massachusetts, United States of America; 2 School of Engineering and Applied Sciences, Harvard University, Cambridge, Massachusetts, United States of America; 3 Wyss Institute For Biological Inspired Engineering, Harvard University, Boston, Massachusetts, United States of America; 4 Department of Biomedical Engineering, Technion - Israel Institute of Technology, Haifa, Israel; Université de Technologie de Compiègne, France

## Abstract

Human embryonic and induced pluripotent stem cells (hESC/hiPSC) are promising cell sources for the derivation of large numbers of specific cell types for tissue engineering and cell therapy applications. We have describe a directed differentiation protocol that generates fibroblasts from both hESC and hiPSC (EDK/iPDK) that support the repair and regeneration of epithelial tissue in engineered, 3D skin equivalents. In the current study, we analyzed the secretory profiles of EDK and iPDK cells to investigate the production of factors that activate and promote angiogenesis. Analysis of *in vitro* secretion profiles from EDK and iPDK cells demonstrated the elevated secretion of pro-angiogenic soluble mediators, including VEGF, HGF, IL-8, PDGF-AA, and Ang-1, that stimulated endothelial cell sprouting in a 3D model of angiogenesis *in vitro*. Phenotypic analysis of EDK and iPDK cells during the course of differentiation from hESCs and iPSCs revealed that both cell types progressively acquired pericyte lineage markers NG2, PDGFRβ, CD105, and CD73 and demonstrated transient induction of pericyte progenitor markers CD31, CD34, and Flk1/VEGFR2. Furthermore, when co-cultured with endothelial cells in 3D fibrin-based constructs, EDK and iPDK cells promoted self-assembly of vascular networks and vascular basement membrane deposition. Finally, transplantation of EDK cells into mice with hindlimb ischemia significantly reduced tissue necrosis and improved blood perfusion, demonstrating the potential of these cells to stimulate angiogenic responses *in vivo*. These findings demonstrate that stable populations of pericyte-like angiogenic cells can be generated with high efficiency from hESC and hiPSC using a directed differentiation approach. This provides new cell sources and opportunities for vascular tissue engineering and for the development of novel strategies in regenerative medicine.

## Introduction

It is well established that mesenchymal progenitor cells make an important contribution to angiogenesis during repair and regeneration. Multiple growth factors regulate the mobilization and recruitment of mesenchymal cells to sites of neovascularization, while directing these cells to a variety of mesenchymal cell fates and functions [1]–[3]. Thus, the biological plasticity of mesenchymal progenitor cells is linked to their ability to promote angiogenesis and vascular regeneration which is essential for defining their therapeutic utility. However, the broad-scale use of mesenchymal cells for regenerative therapies remains somewhat limited due to their heterogeneity *in vivo* that complicates the isolation of well-defined populations of mesenchymal progenitor cells.

The development of functional mesenchymal progenitor cells for specific therapeutic applications has been further complicated by their inherent plasticity. For example, recent studies have suggested that perivascular mesenchymal cells, such as pericytes, may constitute a subset of mesenchymal progenitor cells [4]. It has been shown that the ontogeny of pericytes is complex because they can be traced to various developmental origins including neuroectoderm [5], [6] and mesoderm [7]–[9]. Pericytes do not display definitive molecular markers that can clearly distinguish these cells from other mesenchymal cell types and they share many properties with mesenchymal stem cells (MSCs), including perivascular localization *in vivo*, common molecular markers, and ability to differentiate *in vitro* into various mesenchymal lineages [3], [4], [10], [11]. While pericytes and other stromal cell types of mesenchymal origin play a central role in neovascularization, this uncertainty about their cellular origins and fate currently limit their applications for regenerative therapies.

In light of this, human pluripotent stem cells, such as human embryonic stem cells (hESC) and induced pluripotent stem cells (hiPSC), may be complementary to adult sources of mesenchymal progenitor cells for therapeutic applications. These pluripotent cell sources can be differentiated in ways that direct them to cell types that manifest the functional properties important for angiogenic responses during tissue regeneration. However, the angiogenic potential of hESC- and hiPSC-derived mesenchymal progenitor cells has not been fully explored. Several recent studies have described the isolation of cells with properties overlapping with MSCs from hESC and hiPSC that show several cellular functions that are typical of pericytes [12]–[14]. These cells have been generated upon the spontaneous differentiation of embryoid bodies [12] or by differentiating monolayer cultures of hESC and hiPSC [13], [14]. Cells derived in this way have been shown to stabilize endothelial cell networks *in vitro* and to promote re-vascularization and functional recovery of ischemic tissues *in vivo*. These studies have suggested that these cells are mesenchymal progenitors based on their multilineage differentiation potential, expression of commonly accepted surface markers and their capacity to support angiogenesis both in culture and *in vivo*. However, a more detailed analysis of the ontogeny of these cells has not been explored. In addition, it is not clear whether these previous approaches can generate sufficient numbers of mesenchymal progenitor cells with a proliferative potential that would be useful for clinical applications.

We have recently reported a directed differentiation approach that generates fibroblasts from human pluripotent stem cells using defined substrate and media conditions and exposure to bone morphogenic protein 4 (BMP4) [15], [16] that support epithelial tissue repair and regeneration [17], [18]. We now report that fibroblasts generated using our differentiation protocol from both, hESC and hiPSC (named EDK and iPDK, respectively) support angiogenic processes *in vitro* and *in vivo*. These cells express phenotypic markers of pericytes, secrete angiogenic growth factors and cytokines, promote angiogenesis and formation of vascular network *in vitro,* and rescue limb ischemia *in vivo*. As the critical role of vasculogenesis in tissue repair and regeneration has been elucidated, the innovative use of pluripotent stem cell sources for derivation of angiogenic cells opens new possibilities for disease modeling and for the development of novel strategies for regenerative therapies.

## Results

### EDK and iPDK Cells Secrete Elevated Levels of Pro-angiogenic Growth Factors

**Figure 1:**
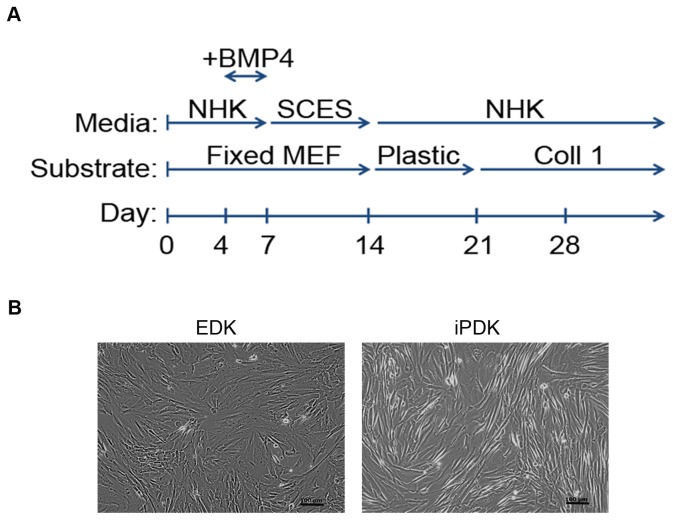
Derivation of EDK and iPDK cells. **A.** Summary of the differentiation protocol used for the derivation of EDK and iPDK cells from hESCs and hiPSCs. **B.** Cells differentiated from hESC (EDK) and hiPSC (iPDK) demonstrate typical fibroblast morphology. Bars, 100 µm.

**Figure 2:**
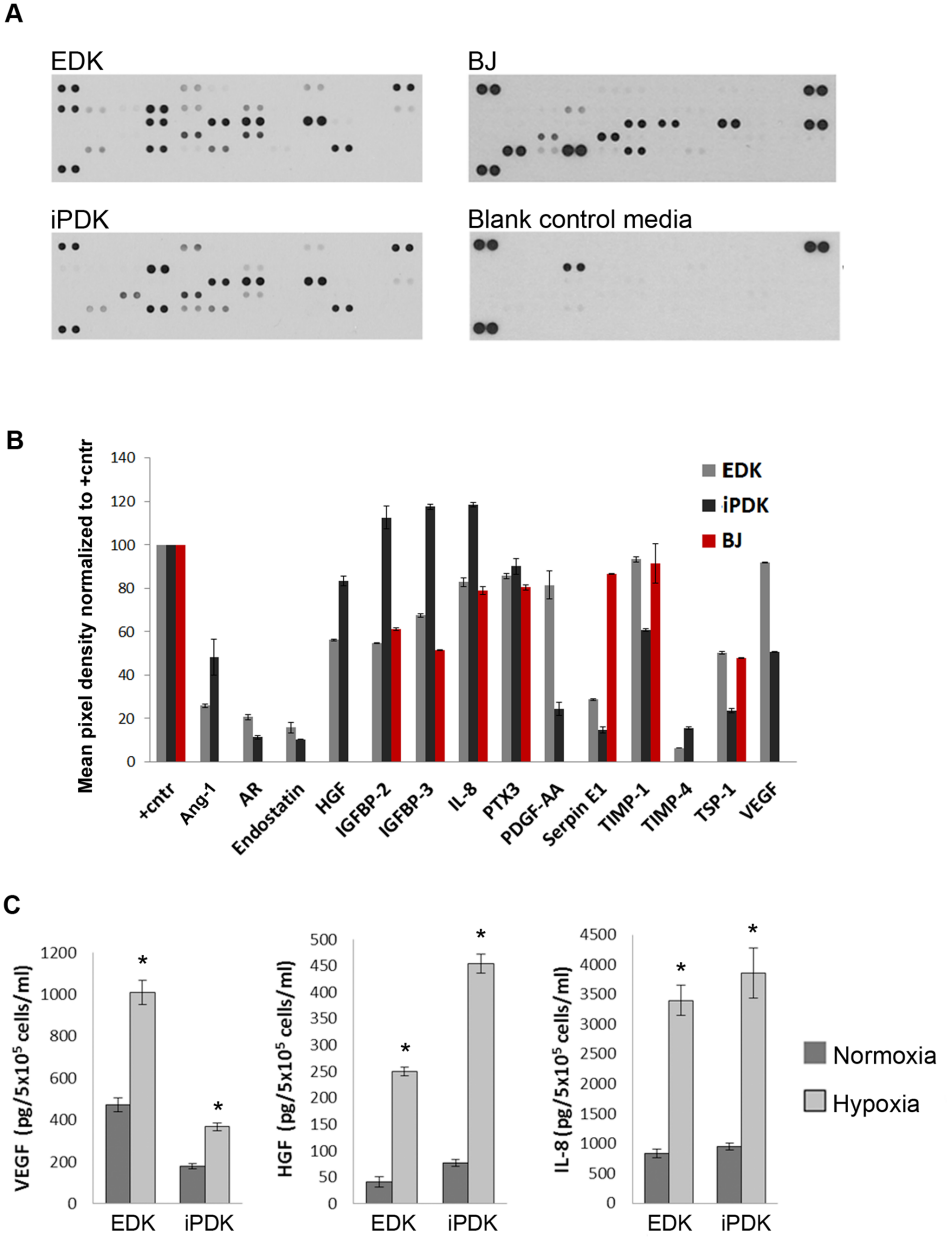
Comparison of secretory protein profiles of EDK, iPDK and BJ cells. **A.** Cytokine array membranes used to analyze the secretion of angiogenic growth factors and cytokines by EDK, iPDK and BJ cells. **B.** Secretory profiles of EDK, iPDK and BJ cells generated from the array membranes. **C.** Expression of selected pro-angiogenic factors VEGF, HGF and IL-8 in response to hypoxia analyzed by ELISA (t-test: *p<0.05).

We first analyzed the secretory profiles of EDK and iPDK cells, that were generated and cultured as previously described [15], [16]. The directed differentiation protocol and the morphology of EDK and iPDK cells are shown in Fig. 1. To identify paracrine factors that may be associated with an angiogenic response, secretory profiles of EDK and iPDK cells were assessed using an antibody array designed to detect soluble mediators of angiogenesis (Fig. 2A). Profiles of EDK and iPDK were generated by quantifying the mean spot pixel densities from the array membranes and normalizing to their respective positive controls (Fig. 2A, 2B, Table 1). EDK and iPDK cells showed very similar secretion profiles (Table 1). Both cell lines expressed elevated levels of pro-angiogenic factors Angiogenin-1 (Ang-1), Amphiregulin (AR), HGF, IGFBP-2, IGFBP-3, IL-8, PDGF-AA, Trombospondin-1 (TSP-1), and VEGF, as well as anti-angiogenic factors Endostatin, PTX3, and Serpin E1 (Fig. 2B). In addition, both EDK and iPDK showed elevated levels of matrix metalloproteinase regulators TIMP1 and TIMP4, which are known to play a role in proteolytic matrix degradation during angiogenic sprouting (Fig. 2B). There was a noticeable difference in levels of Coagulation factor III (TF), CXCL16, KGF, MCP-1, and MMP9 in EDK when compared to iPDK cells (Table 1). Analysis of the secretory profile of control, dermal-derived fibroblasts (BJ) using the same antibody array showed that the levels of key angiogenic regulators VEGF, PDGF-AA, HGF, Ang-1, AR, Endostatin, and TIMP-4 were significantly lower when compared to secretory profiles of EDK and iPDK cells (Fig. 2A, 2B, Table 1). We then confirmed the expression levels of select pro-angiogenic factors VEGF, HGF and IL-8 using ELISA and found that the production of these factors was dramatically induced by hypoxia in both EDK and iPDK cells (Fig. 2C). These findings demonstrated that cells derived from hESC and hiPSC secrete a similar spectrum of paracrine factors known to stimulate angiogenesis.

**Table 1:** Secretory profiles of EDK, iPDK and BJ cells.

	EDK		iPDK		BJ	
Target/Control	mean	stdv	mean	stdv	mean	stdv
**control (+)**	100.00		100.00		100.00	
**Angiogenin-1 (Ang-1)**	25.93	0.74	48.13	8.31	0.00	0.00
**Amphiregulin (ER)**	20.55	1.06	11.30	0.64	0.00	0.00
**Coagulation factor III (TF)**	44.82	0.25	3.98	0.68	0.00	0.00
**CXCL16**	7.44	0.74	1.11	0.00	0.00	0.00
**EG-VEGF**	12.48	0.02	1.00	0.00	0.00	0.00
**Endostatin**	15.74	2.52	10.42	0.00	0.00	0.00
**FGF-7 (KGF)**	6.55	0.95	1.13	0.11	0.00	0.00
**HGF**	56.14	0.30	83.19	2.13	0.00	0.00
**IGFBP-2**	54.82	0.21	82.36	5.18	60.97	0.65
**IGFBP-3**	67.50	0.85	87.27	1.04	51.25	0.19
**IL-8**	82.86	2.03	88.31	0.98	78.91	1.95
**MCP-1**	0.97	0.11	8.83	0.17	85.56	3.84
**MMP9**	3.22	0.44	70.36	4.00	31.57	1.12
**PTX3**	85.58	1.17	90.05	3.56	80.32	1.18
**PDGF-AA**	81.40	6.44	24.44	2.96	0.00	0.00
**Serpin E1**	28.70	0.38	14.70	1.28	86.45	0.16
**TIMP-1**	93.18	1.21	60.72	0.77	91.41	9.00
**TIMP-4**	3.34	0.06	15.54	0.55	0.00	0.00
**Thrombospondin-1** **(TSP-1)**	50.21	0.59	23.61	0.94	47.81	0.23
**VEGF**	91.73	0.25	50.69	0.30	0.00	0.00

Supernatants from EDK and iPDK cultures containing equal cell numbers and blank control media were harvested and assayed using an antibody-based cytokine array. Secretory profiles were generated by quantifying the mean spot pixel densities by ImageJ from the array membranes shown in Fig. 2A. The data are presented as percentages of the respective positive controls.

### EDK and iPDK Cells Express Phenotypic Markers of Pericytes

**Figure 3:**
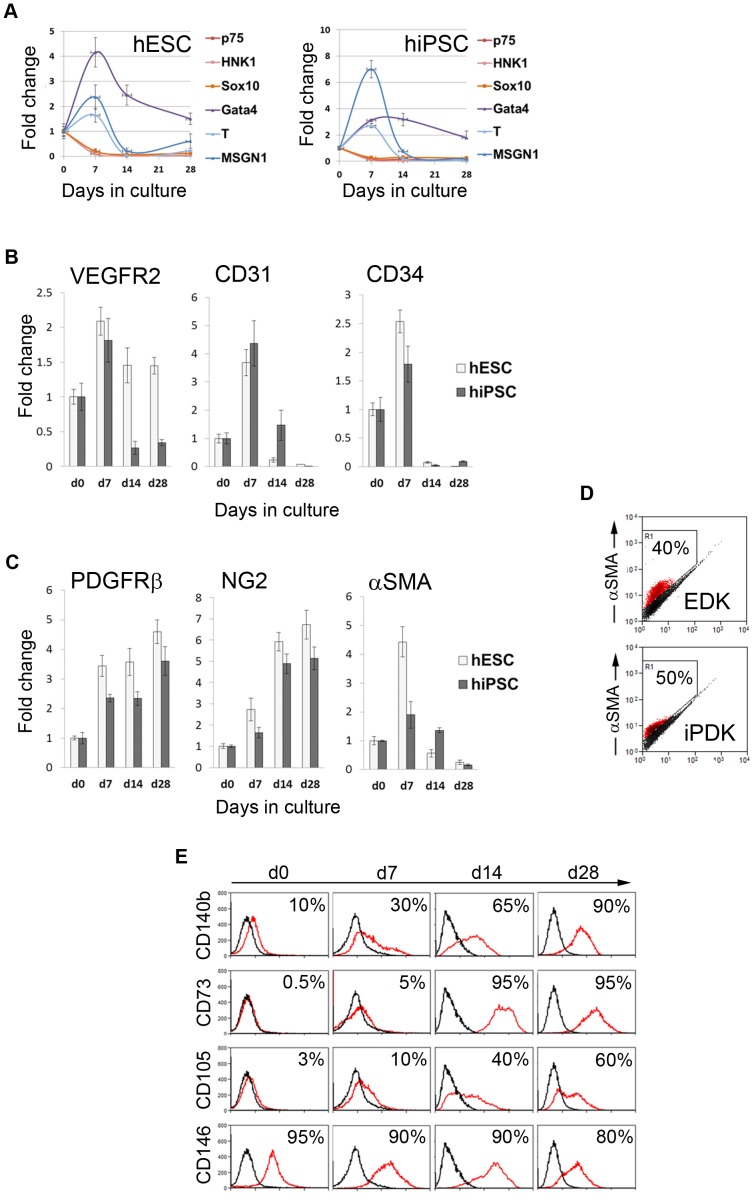
Emergence of pericyte markers following differentiation from hESC and hiPSC. **A.** Real time RT-PCR analysis of neural crest and mesodermal markers following differentiation from hESC and iPSC at day 0, 7, 14, and 28 of differentiation. **B.** Real time RT-PCR analysis of vasculogenic precursor markers following differentiation from hESC and iPSC at day 0, 7, 14, and 28 of differentiation. **C.** Real time RT-PCR analysis of pericyte markers following differentiation from hESC and iPSC at day 0, 7, 14, and 28 of differentiation. **D.** Flow cytometric analysis of αSMA protein expression in differentiated EDK and iPDK cells. **E.** Flow cytometric analysis of protein levels of pericyte markers CD140b, CD73, CD105, and CD146 in hESC at day 0, 7, 14, and 28 of differentiation (marker expression - red profiles are shown relative to isotype control - black profiles).

To learn more about the lineage of EDK and iPDK cells, these cells were monitored for expression of neural crest and mesodermal progenitor markers during sequential stages of differentiation. The change in mRNA and protein expression following induction of differentiation of hESC and hiPSC was analyzed at days 0, 7, 14, and 28 days by real-time RT-PCR and flow cytometry. Data analysis revealed similarities in temporal gene expression profiles of differentiating hESC and hiPSC, which showed downregulation of neural crest markers p75(NTR), HNK1 and Sox10 and transient induction of mesodermal progenitor markers GATA4, T (Brachyury homolog) and Mesogenin-1 (MSGN1) following BMP4 treatment at day 7 (Fig. 3A). These results indicate that culture conditions supporting the differentiation of hESC and hiPSC towards mesodermal progenitors and the divergence from neuroectodermal fate. Further analysis revealed the transient induction of markers associated with vasculogenic progenitors, including VEGFR2, CD34 and CD31 following BMP4 treatment at day 7 of differentiation (Fig. 3B). Analysis of markers associated with pericytes showed a gradual increase in expression levels of NG2, PDGFRβ, CD73, and CD105 (Fig. 3C, 3E, Table 2). Analysis of levels of CD146 showed a slight decrease throughout the differentiation of hESC and hiPSC, from 95% to 75% and 95% to 80%, respectively (Fig. 3E, Table 2). The gene expression level of αSMA was increased at day 7 of differentiation, however this level decreased significantly by day 14, and was 5-fold downregulated at day 28 compared to pluripotent cells (Fig. 3C). Flow cytometric analysis of αSMA protein showed that 40% of differentiated EDK and 50% of iPDK cell population stained positively for this protein (Fig. 3D), demonstrating that αSMA was sustained in these cells. Taken together, temporal gene and protein expression patterns suggest that culture conditions were conducive for differentiation of hESC and iPSC towards a lineage with features overlapping with pericytes.

**Table 2:** Flow cytometric analysis of surface markers characteristic of pericytes.

			% of positive cells			
Surface marker	Antigen	Celltype	d0	d7	d14	d28
**CD140b**	PDFGRβ	hESC	10%	30%	65%	90%
		hiPSC	30%	50%	60%	90%
**CD73**	SH3, ecto-5′-nucteotidase	hESC	0.5%	5%	95%	95%
		hiPSC	5%	–	–	99%
**CD105**	SH2, endoglin	hESC	3%	10%	40%	60%
		hiPSC	10%	–	–	90%
**CD146**	MCAM	hESC	95%	90%	90%	80%
		hiPSC	95%	–	–	75%

Human ESC and hiPSC were induced to differentiate in parallel using identical differentiation procedures and the expression of CD73, CD105, CD146, and CD140b during the sequential stages of differentiation was analyzed by flow cytometry. The percentages of cells positive for the cell surface markers are shown. Each experiment is normalized to isotype control, and has been repeated at least 2 times.

### Angiogenic Factors Secreted by EDK and iPDK Cells Induce Endothelial Sprouting *in vitro*

**Figure 4:**
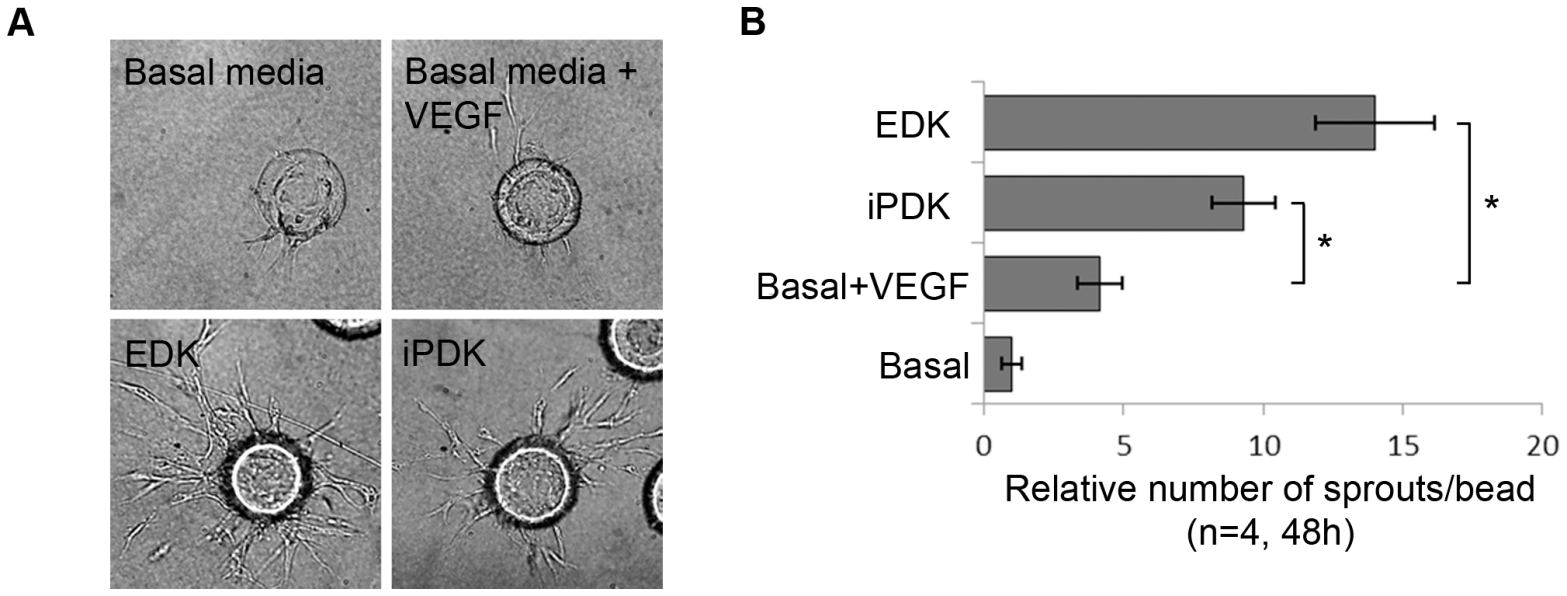
Angiogenic factors secreted by EDK and iPDK cells promote endothelial cell sprouting. **A.** Representative images of endothelial sprouts formed in EDK- and iPDK-containing cultures and control cultures. **B.** Quantification of endothelial sprouts in EDK- and iPDK-containing cultures and control cultures (t-test: *p<0.05).

To determine whether soluble factors produced by EDK and iPDK cells could promote endothelial sprouting, these cells were incorporated into a 3D, *in vitro* sprouting assay that recapitulates the early stage of the angiogenic process [19]. For this assay, microcarrier beads were coated with human dermal-derived microvascular endothelial cells (HMVEC) and embedded into a fibrin gel. EDK and iPDK cells were then layered on the gel surface to test if their secretion of soluble factors could promote endothelial cells sprouting from the surface of the beads. After incubation for 48 hours, numerous sprouts were seen in EDK- and iPDK-containing cultures compared to control cultures grown in basal media or basal media supplemented with 50 ng/ml of VEGF (Fig. 4A). VEGF supplementation led to a slight increase in sprouting when compared to levels seen for incubation with basal media (Fig. 4A). Quantification of endothelial sprouts revealed that their number was significantly increased in both EDK- and iPDK-containing cultures when compared to both control cultures (Fig. 4B). These findings suggest paracrine mechanisms are linked to the activation of endothelial cell sprouting by EDK and iPDK cells.

### EDK and iPDK Cells Support 3D Vascular Network Formation *in vitro*

**Figure 5:**
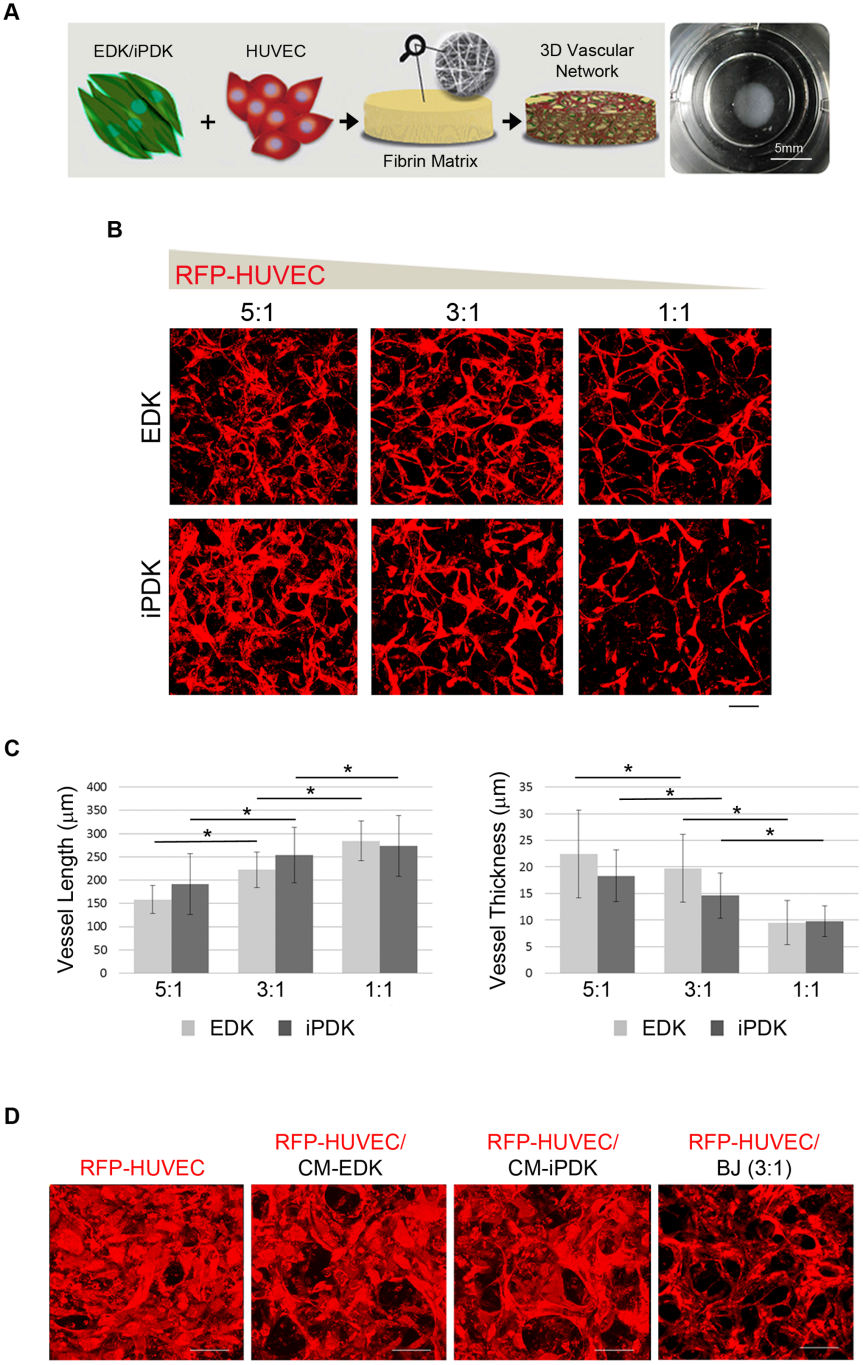
Endothelial cells co-cultured with EDK and iPDK form vascular networks. **A.** Schematic showing engineering of 3D vascular network *in vitro* and an image of a fibrin construct (typical dimensions: 5 mm diameter and 0.25 mm height). **B.** Representative confocal images of 3D vascular networks formed within fibrin matrix following seeding of RFP-HUVEC mixed with EDK and iPDK at ratios of 5∶1, 3∶1, and 1∶1 (collapsed Z-stacks of total 250±25 µm). Bar, 100 µm. **C.** Quantification of vessel length and thickness of 3D vascular networks formed within fibrin constructs (ANOVA: *p<0.05). **D.** Representative confocal images of RFP-HUVEC cultured alone within fibrin matrix in basal media, in EDK- and iPDK-conditioned media, or co-cultured with BJ at ratio 3∶1 at day 8 post-seeding (collapsed Z-stacks of total 65±5 µm). Bars, 100 µm.

As another functional outcome linked to angiogenesis, we next studied the ability of EDK and iPDK cells to support *in vitro,* vascular network formation within 3D fibrin-based constructs (Fig. 5A). RFP-expressing human umbilical vein endothelial cells (RFP-HUVEC) were mixed with either EDK or iPDK cells at ratios of 5∶1, 3∶1 and 1∶1 within fibrin matrices, and allowed to spontaneously assemble into vessel-like networks for 8 days. Confocal microscopy analysis showed that after 8 days, RFP-HUVEC cells formed interconnected vessel-like networks in the presence of both EDK and iPDK cells at all ratios of RFP-HUVEC: EDK and RFP-HUVEC: iPDK tested (5∶1, 3∶1 and 1∶1) (Fig. 5B). Assessment of network morphology revealed a significant increase in mean vessel length and a decrease in vessel thickness as the ratio of RFP-HUVEC to EDK and iPDK decreased (Fig. 5C). In contrast, RFP-HUVEC cultured alone in complete endothelial media or in the media conditioned by EDK or iPDK cells for 24 hours failed to form interconnected vascular networks (Fig. 5D). Foreskin-derived BJ fibroblasts co-cultured with RFP-HUVEC at ratio 3∶1 could promote a minor degree of patterning of endothelial cells but failed to induce formation of capillary-like structures of uniform length or diameter as seen for EDK- and iPDK-containing cultures (Fig. 5D). This indicates that EDK and iPDK cells provide a specific set of signals to drive the formation of stable capillary-like network.

### EDK and iPDK Cells are Required for Stabilization of 3D Vascular Network and Deposition of Vascular Basement Membrane

**Figure 6:**
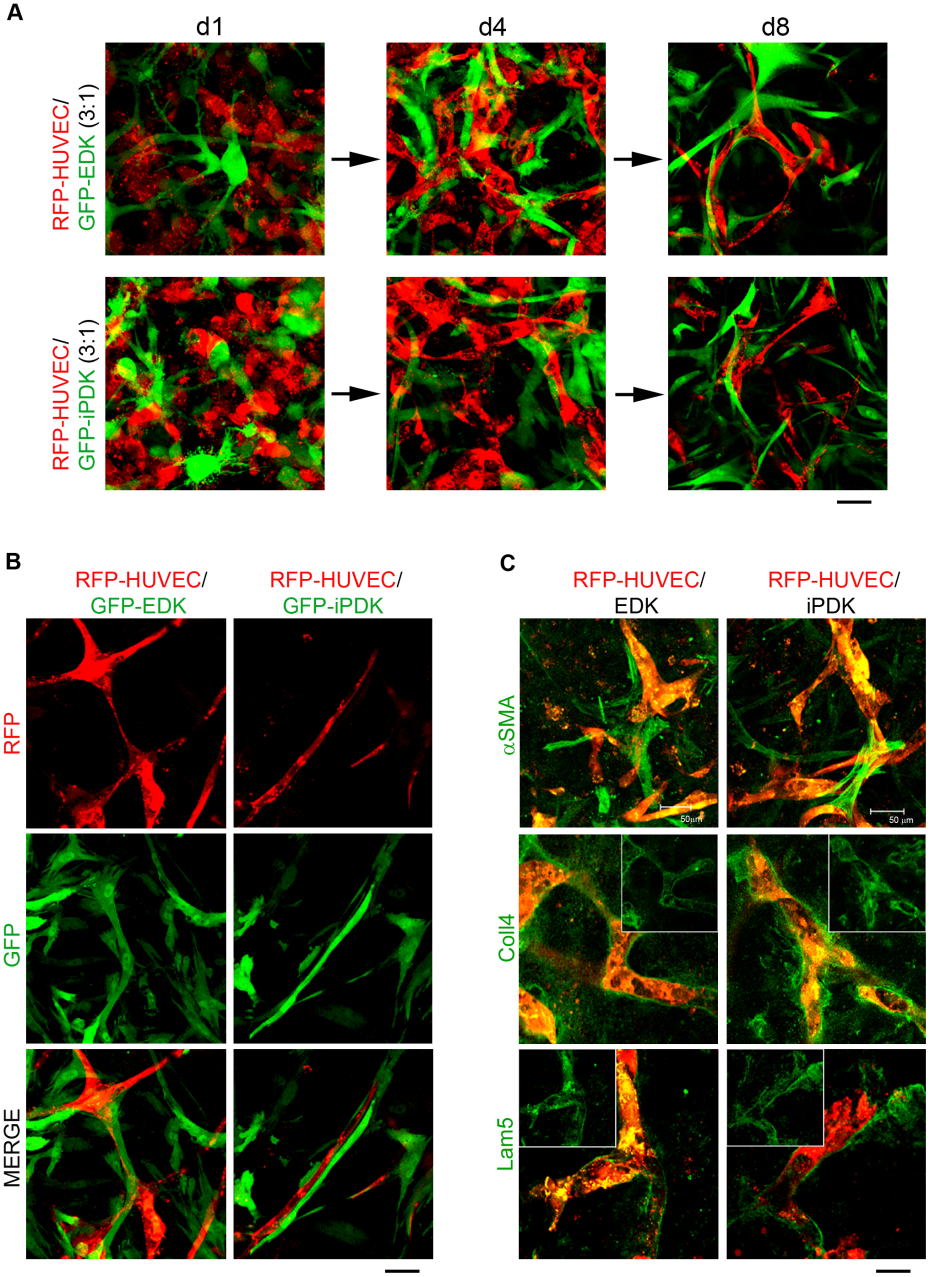
Fate of EDK and iPDK cells within fibrin-based constructs and level of vessel maturity. **A.** Organization of RFP-HUVEC and GFP-expressing EDK and iPDK cells within 3D fibrin constructs over time. Representative confocal images of 3D vascular networks formed within fibrin constructs at day 1, 4, and 8 co-cultures (collapsed Z-stacks of total 250±25 µm). Bars, 50 µm. **B.** Confocal images taken at higher magnification illustrating EDK and iPDK cells co-localizing with vascular networks at day 8 co-cultures (collapsed Z-stacks of total 15±2 µm). Bars, 25 µm. **C.** Confocal imaging analysis of 3D vascular networks formed with RFP-HUVEC (red) and EDK and iPDK cells immunostained with αSMA (bars, 50 µm), Coll4 and Lam5 (all green) at day 8 co-cultures (collapsed Z-stacks of total 15±2 µm). Bars, 25 µm.

In order to obtain further insights into interactions between pluripotent stem cell-derived fibroblasts and endothelial cells, EDK and iPDK cells were labeled with GFP and incorporated into fibrin constructs with RFP-HUVEC at a ratio 3∶1 (HUVEC:EDK/iPDK). Fibrin constructs were monitored for 8 days after seeding using confocal microscopy. During this 8 day period, RFP-HUVEC co-cultured with GFP-EDK and GFP-iPDK underwent phenotypic changes that resulted in formation of interconnected vascular networks (Fig. 6A). After 1 day in culture, GFP-EDK and GFP-iPDK cells began spreading in the matrix while RFP-HUVEC remained rounded (Fig. 6A, 1d). After 4 days in culture, RFP-HUVEC became elongated and formed disorganized partially connected vascular networks that co-localized with GFP-expressing EDK and iPDK cells situated at various points above and below the focal plane (Fig. 6A, 4d). After 8 days in culture, RFP-HUVEC assembled into stable vascular networks with segments of uniform diameter (∼15–20 µm) that co-localized with GFP-expressing, EDK and iPDK cells (Fig. 6A, 8d, Fig. 6B, Video S1). As neither monoculture of RFP-HUVEC in complete endothelial media nor monoculture of RFP-HUVEC in EDK- and iPDK-conditioned media resulted in formation of interconnected vascular networks (Fig. 5D), direct interactions between EDK and iPDK cells are likely to be necessary to promote organization and stabilization of vascular networks by endothelial cells, suggesting a possible pericyte function for these cells. Since the formation of the vascular basement membrane is a hallmark of vessel maturation [20], we next analyzed the deposition of vascular basement membrane matrix in 3D fibrin-based constructs prepared with RFP-HUVEC and EDK and iPDK cells 8 days after seeding. Confocal whole mount immunofluorescence analysis of fibrin constructs stained with antibodies specific to Type IV Collagen (Coll4) and Laminin subunit α5 (Lam5), main constituents of vascular basement membrane, revealed Coll4 and Lam5 deposition at the interface between RFP-expressing HUVEC and EDK and iPDK cells that enveloped the vascular networks (Fig. 6C). In addition, immunofluorescence staining of αSMA revealed abundant αSMA-positive EDK and iPDK cells in direct contact with RFP-HUVEC (Fig. 6C). These results indicated that EDK and iPDK cells supported the deposition of vascular basement membrane matrix as well as the organization of 3D vascular networks that suggest a pericyte-like function in our *in vitro* models.

### EDK Cells Reduced Tissue Necrosis and Improved Blood Perfusion when Transplanted in a Murine Model of Limb Ischemia

**Figure 7:**
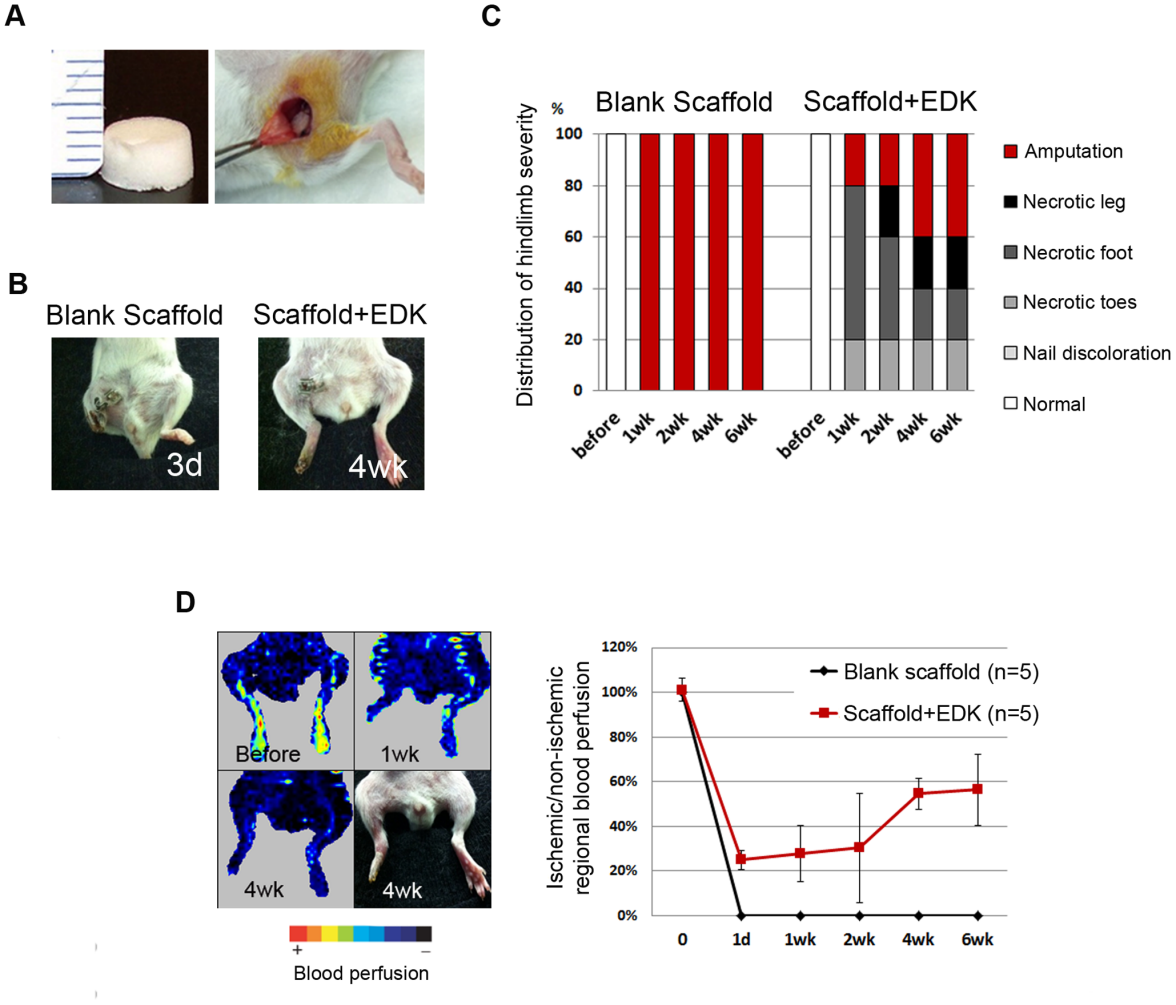
Transplantation of EDK cells into ischemic hindlimbs. **A.** Images demonstrating the transplantation of alginate scaffold seeded with EDK cells into ischemic hindlimbs. **B.** Representative images of an ischemic limb transplanted with either control blank scaffold at 3 days post-transplantation or with scaffold seeded with EDK cells at 4 weeks post-transplantation. **C.** Distribution of ischemic severity given by visual examination of ischemic limbs following cell transplantation as a function of time post-surgery. **D.** Laser Dopler Perfusion images of ischemic hindlimbs after treatment with EDK cells and quantitative analysis of hindlimb blood flow after treatment with EDK cells.

To analyze whether hESC-derived mesenchymal cells could stimulate angiogenic responses *in vivo*, we transplanted EDK cells into a murine model of hindlimb ischemia that was created by femoral artery and vein ligation in SCID mice. RGD-coupled alginate scaffolds (Fig. 7A) were utilized as a cell delivery vehicle to transplant EDK cells into ischemic areas to support their host integration [21]. Blank alginate scaffolds (n = 5) and alginate scaffolds seeded with 0.5×10^6^ of EDK cells (n = 5) were transplanted subcutaneously into the area of femoral artery ligation (Fig. 7B) and the mice were followed for 6 weeks post-transplantation. Hindlimbs subjected to surgery were visually examined at 1 and 3 days and 1, 2, 4 and 6 weeks after surgery and scored based on the evaluation of degree of necrosis (5 = normal 4 = presenting nail discoloration, 3 = multiple necrotic toes, 2 = necrotic foot, 1 = necrotic leg, 0 = complete amputation). Visual examination of hindlimbs revealed that mice treated with blank scaffolds rapidly suffered from necrosis and loss of the ischemic hindlimbs within three days after surgery, and could not be used for any further analysis (Fig. 7B, 7C). In contrast, transplantation of EDK cells greatly limited such autoamputation and the overall degree of necrosis was reduced (Fig. 7B, 7C). By 4 weeks after surgery, ischemic hindlimbs in EDK group stabilized at either the necrotic foot or necrotic leg stage (Fig. 7C). To analyse the perfusion of ischemic hindlimb after cell transplantation, Laser Dopler Perfusion Image (LDPI) analysis was performed at 1 day and 1, 2, 4 and 6 weeks after surgery. Femoral artery and vein ligation led to a rapid loss of perfusion in the ligated limbs as animals treated with blank scaffolds demonstrated rapid limb necrosis and no perfusion images were obtained. Animals treated with EDK cells demonstrated rapid loss of blood perfusion linked to induction of hindlimb ischemia (∼30% of normal perfusion levels), which stayed constant for 2 weeks following surgery (Fig. 7D). By 4 weeks, significant blood flow recovery was observed in EDK group reaching ∼60% of normal perfusion levels (Fig. 7D). These findings demonstrated that transplantation of EDK cells activated angiogenic responses *in vivo*.

## Materials and Methods

### Cell Culture

Adult dermal-derived human blood microvascular endothelial cells (HMVEC) were purchased from Lonza Inc. (Lonza, Basel, CH). Human umbilical vein endothelial cells (HUVEC) and RFP-expressing HUVEC (RFP-HUVEC) were purchased from Angio-Proteomie (Angio-Proteomie, Boston, USA). All endothelial cells were expanded and maintained on tissue culture plastic in EGM-2MV media (Lonza, Basel, CH). EDK and iPDK cell lines were differentiated from the H9 hESC cell line and an iPSC line derived from BJ fibroblasts as previously described [15], [16]. The H9 line of hESC was purchased from the WiCell Institute (Madison, WI). For all experiments, EDK and iPDK cell lines were grown on Type I Collagen-coated plates (BD Biosciences, San Jose, CA) in NHK media consisting of 3∶1 DMEM:F12 (Invitrogen, Carlsbad, CA), 5% FCII (Hyclone, Logan, UT), 0.18 mM adenine, 8 mM HEPES, 0.5 µg/mL hydrocortisone, 10^−10^ M cholera toxin, 10 ng/mL EGF, 5 µg/mL insulin (all from Sigma, St. Louis, MO). All cell lines were routinely checked for mycoplasma contamination using MycoAlert® Mycoplasma detection kit (Lonza, Rockland, ME).

### Flow Cytometry

Cells were trypsinized, pelleted, and re-suspended in 2% FBS in PBS. Cell suspensions were stained with PE-conjugated anti-CD73, -CD105, -CD140b, -CD146 - or Isotype control-IgG1k (BD Pharmingen, San Jose, CA) for 30 min on ice. For αSMA staining, cells were trypsinized, pelleted, and fixed in 0.01% paraformaldehyde, permeabilized using 0.5% Saponin, and stained with anti-αSMA primary antibodies (Abcam, Cambridge, MA) followed by Alexa Fluor 594-conjugatedgoat anti-mouse secondary antibody (Invitrogen, Carlsbad, CA). All data were acquired using a FACSCalibur (BD, San Jose, CA) and analyzed using CellQuest (BD, San Jose, CA) and Summit V4.3 software (Dako, Carpentaria, CA). Analysis was performed on 20,000 cells per sample and results are representative of two independent experiments.

### Real-time RT-PCR

RNA was isolated using QiagenRNeasy purification kit (Qiagen, Valencia, CA), and then converted to cDNA with the iScriptcDNA synthesis kit (Biorad, Hercules, CA) using 0.5 µg RNA. PCR reactions were done in triplicate with 2× SYBRgreenSupermix (Biorad, Hercules, CA) and run on a iQ5 Real-Time PCR detection system (Bioad, Hercules, CA). Error bars represent standard deviation of 3 biological replicates. The primer sequences for the genes used in this study are listed in Table S1.

### Antibody-based Cytokine Array

EDK and iPDK 10^6^ cells were plated onto 100 mm plates in tissue culture media and grown to 80–90% confluence. All cells were fed with 5 ml of fresh tissue culture media 24 hours prior the experiment. Tissue culture supernatants were harvested, and the supernatants from the plates containing equal cell numbers were processed using Proteome Profiler Human Angiogenesis Antibody Array (R&D Systems, Minneapolis, MN) according to manufacturer’s protocol. Histogram profiles were generated by quantifying the mean spot pixel densities from the array membranes using ImageJ software (U. S. National Institutes of Health, Bethesda, Maryland, USA) and plotted as percentages of the respective internal positive controls. For angiogenesis array alignment and coordinates see Table S2.

### ELISA

Cells were grown either in normoxic or hypoxic conditions (1% O_2_) for 48 hours. Tissue culture supernatants were harvested and processed using DuoSetVEGF, HGF and IL-8 ELISA kits (R&D Systems, Minneapolis, MN) according to manufacturer’s protocol. Media was assayed in triplicates from at least three independent samples. The values were normalized according to cell numbers counted in the respective cultures at the time of supernatant harvesting and expressed in pg/ml per 10^4^ cells.

### 3D In Vitro Sprouting Assay

3D *in vitro* sprouting assay was performed using HMVEC-coated dextran beads embedded in fibrin gel in a 24-well plate as previously described [22]. The composition of the fibrin gel in each well was 0.68 mg fibrinogen, 11.4 µg aprotinin, 0.455 U thrombin (all from Sigma, St. Louis, MO) in 393 µL of PBS and 57 µL of basal EGM-2MV. The basal EGM-2MV media containing VEGF (50 ng/ml), or the basal EGM-2MV media containing EDK or iPDK cells (0.5×10^4^cells/cm^2^) were added into each well and incubated for 48 h. After 48 h, the gels were fixed with 4% paraformaldehyde and visualized with an Olympus IX2 microscope. Sprouts were identified as continuous multi-cellular structures extended from the microcarrier beads with a minimum of two cells in the structure.

### Preparation of 3D Vascular Networks

3D vascular networks were prepared as previously described [23]. RFP-HUVEC 0.3×10^6^, 0.15×10^6^, or 0.06×10^6^and P-EDK or P-iPDK 0.06×10^6^ were mixed together, pelleted, and resuspended with 60 µl of fibrin solution. The composition of the fibrin solution in each was 7 mg/ml fibrinogen, 50 µg/ml aprotinin, and 20 U/ml thrombin (all from Sigma, St. Louis, MO). Mixtures were pipetted into glass-bottom 12-well plates and incubated at 37°C for 15 min to gel. EGM-2MV media (Lonza, Basel, CH) was added into each well and incubated for 7 h. Media were changed every other day.

### GFP Labelling of EDK and iPDK Cells

Lentiviral particles carrying GFP (gift of the lab of Dr. Larry Feig. Tufts University) were generated in 293FT cells using ViraPower™ Lentiviral Expression System (Invitrogen, Carlsbad, CA) according to manufacturer’s protocol. The pLenti-CMV-GFP-puro vector was purchased from Addgene (Addgene, Cambridge, MA). GFP-P-EDK and GFP-P-iPDK cell lines were generated by infection of 50,000 cells with 1 MOI of lentivirus carrying GFP sequence. Stable cell lines were selected with puromycin (2 µg/ml) (Sigma, St. Louis, MO).

### Immunofluorescence and Confocal Imaging

3D fibrin constructs were scanned by Leica™ TCS SP2 confocal microscope after 1, 4, and 8 days in culture. Laser excitation wavelengths included 488 nm and 561 nm. Emission spectrum was freely tunable between 425 nm and 740 nm. Sample scanning was recorded every 10 µm for ×20 magnification, and every 1 µm for ×40 magnification. For immunofluorescence, 3D fibrin constructs were fixed with 4% paraformaldehyde for 10 min, permeabilized using 0.3% Triton X-100 for 10 min, and immersed overnight in blocking solution containing 10% goat serum and 0.1% Triton X-100 at 4°C. Constructs were stained overnightwith anti-Type IV Collagen(Sigma, St. Louis, MO), Laminin5 (Millipore, Billerica, MA), and αSMA (Abcam, Cambridge, MA) primary antibodies followed by 3 h incubation with Alexa Fluor 488 -conjugated goat anti-mouse secondary antibodies (Invitrogen, Carlsbad, CA).

### Quantification of 3D Vascular Networks

3D fibrin constructs were scanned by Leica™ TCS SP2 confocal microscope after 8 days in culture using ×20 lens generating 25±2.5 optical slices of 10 µm each. The resulting image stacks of RFP-HUVEC were subjected to a series of image analyses using SPOT Advanced software (Diagnostic Instruments, Sterling Heights, MI). Vessel length was calculated between proximity branches. Vessel thickness was calculated in the middle of each sprout. Two independent constructs and three z-stacks of images taken at different focus depths per construct were analyzed for each condition.

### Preparation of RGD-coupled Alginate Scaffolds

Alginate scaffolds were prepared using high molecular weight (∼250 kDa) ultrapure sodium alginate powder (Novamatrix Pronova UP MVG alginate) enriched (≥60%) in G blocks as previously described [21]. Briefly, a 2% w/v alginate solution in dH_2_O was oxidized to 1% with sodium periodate to create hydrolytically labile bonds in the polymer. Oxidized alginates were coupled with oligopeptides containing the Arg-Gly-Asp cell adhesion sequence (Commonwealth Biotechnologies, Richmond, VA) following aqueous carbodiimide chemistry. Hydrogels were prepared by mixing the alginate solution with calcium sulfate slurry and the mixture was injected between glass plates with a spacer of 1 mm. After curing for 20 min, gel disks with diameter of 10 mm were punched out. These gel disks were frozen and stored at −20°C, and after 24 h, gel disks were lyophilized to yield macroporous materials.

### Ischemic Hindlimb Model in SCID Mouse

All procedures were carried out at Harvard University and were approved by the Experimental Animal Committee of Harvard University. SCID mice were subjected to femoral artery and vein ligation to induce hindlimb ischemia. Immediately after ligation cell-loaded alginate scaffolds (5×10^6^ cells per scaffold) or control blank scaffolds were transplanted on the medial side of thigh muscle. Before the surgery (day 0), and 1 day, 7 days, 2, 4 and 6 weeks postsurgery, hindlimbs subjected to surgery were visually examined, and each received a score based on the evaluation of the degree of necrosis. Measurements of the ischemic/normal limb blood flow ratio were performed on anesthetized animals (*n* = 5/time point/experimental condition) using a Periscan system blood perfusion monitor laser Doppler equipment (Perimed Instruments, Ardmore, PA).

### Statistical Analysis

Statistical analyses were carried out using IBM SPSS Statistics 19 software (IBM, Armonk, NY). All results are reported as mean ± standard deviation of at least three independent samples. Statistical comparison between two groups was performed using Student’s t-test. When comparing more than two groups One-Way Analysis of Variance (ANOVA) test was used followed by the post hoc Tukey’s multiple comparison tests. Results were considered significant for p≤0.05.

## Discussion

Cell-based therapies are currently generating a great deal of interest to more effectively treat ischemic diseases. Previous studies have shown that the most effective strategies to promote neovascularization involve co-delivery of endothelial cells with supporting stromal cells that control functional properties of the developing capillaries [24]. However, neither an optimal source nor optimal type of stromal cells that support angiogenic responses has been defined so far. In light of this, human pluripotent stem cells, such as hESC and hiPSC, provide plentiful and renewable cell sources for derivation of stromal cell types that manifest properties important for the induction of angiogenic responses during tissue regeneration.

There are a number of studies that have shown the derivation of MSC-like cells from human pluripotent stem cells that express phenotypic markers of pericytes, support formation of endothelial networks *in vitro* and promote re-vascularization of ischemic tissues *in vivo* [12]–[14]. We have extended these findings to demonstrate that fibroblasts derived from hESC and hiPSC (EDK and iPDK, respectively) using our differentiation protocol manifest a more diverse repertoire of cellular features that support angiogenesis. Comparative studies of EDK and iPDK cells demonstrated that both, hESC and hiPSC-derived fibroblasts show similar patterns of gene expression, secretion of pro-angiogenic growth factors and functional angiogenic outcomes. This suggests that our directed differentiation approach allows differentiation of angiogenic stromal cells from human pluripotent stem cells with high reproducibility and efficiency.

Functional recovery of ischemic tissue, such as an ischemic limb and heart by pluripotent stem cell-derived mesenchymal progenitor cells have been previously reported [12], [13], [25], [26]. However, the mechanism by which these cells may contribute to the repair of ischemic tissues remains somewhat controversial. One possibility is that multipotent progenitor cells are capable of incorporating into mature vasculature by differentiating into pericytes and smooth muscle cells. A second option is that the various stromal cell types secrete distinct pro- and anti-angiogenic cytokines that enhance angiogenesis and augment blood flow to ischemic tissue. Although the homing of transplanted hESC-derived MSCs into ischemic tissues has been reported [26], the findings of low proliferation and the limited survival of transplanted cells appear to support the paracrine hypothesis. Several studies have demonstrated the paracrine effects of adult tissue-derived MSC including bone-marrow and adipose tissue-derived MSCs, both *in vitro* and *in vivo* [27]–[32]. For example, pro-angiogenic growth factors and cytokines, such as VEGF, HGF, bFGF, Ang-1, IL-6, IL-8, MCP-1 and others, have been identified within *in vito* secretome of adult tissue-derived MSCs [27], [28], [32]. Paracrine contribution of adult tissue-derived MSC *in vivo* has also been demonstrated in a murine model of hind limb ischemia [27], where MSCs promoted the recovery of ischemic limb through secretion of pro-angiogenic growth factors, specifically VEGF that was detected *in situ* around the transplanted cells [27]. However, the paracrine effects of hESC- and hiPSC-derived stromal cells have not been fully explored.

To gain insight into the mechanism by which hESC- and iPSC-derived fibroblasts may contribute to angiogenic responses, we analyze the secretion profiles of EDK and iPDK cells *in vitro*, by focusing on factors that activate and promote angiogenesis. Importantly, both EDK and iPDK cells demonstrated very similar secretory profiles of angiogenic factors showing elevated levels of VEGF, Ang-1, PDGF-AA, HGF, AR, Endostatin, and TIMP-4 when compared to control, BJ fibroblasts. These findings indicate that these paracrine factors may play an important role in angiogenic process mediated by EDK and iPDK cells. Indeed, when incorporated into 3D *in vitro* angiogenesis assay in a medium devoid of growth factors, angiogenic factors secreted by EDK and iPDK cells induced sprouting of microvascular endothelial cells (HMVEC), thus indicating their potential to activate therapeutic angiogenesis. Beyond this, transplantation of EDK cells to ischemic hindlimbs demonstrated similar injury responses that were characterized by increased blood perfusion over time and partial recovery of ischemic limbs. These results demonstrated the potential of hESC-and hiPSC-derived fibroblasts to induce neovascularization *in vivo*. Additional *in vivo* studies will be necessary to further establish the role that EDK and iPDK play in this vascular response.

In order to explore the correlation between developing origin of EDK and iPDK cells and phenotype to angiogenic properties, we analyzed the ontogeny of these cells following their differentiation from pluripotent stem cells. Analysis of the ontogeny of these cells revealed markers that are reminiscent of pericytes, including NG2, PDGFRβ, αSMA, CD73, and CD105, that may help explain the angiogenic outcomes we found *in vitro* and *in vivo*. We found the expression of these markers shortly after BMP4 treatment that occurred concomitant with the transient induction of FLK1/VEGFR2^+^, CD34^+^ and CD31^+^. We have shown that expression of the pericyte marker CD146 was not dependent on BMP4 treatment and remained stable throughout differentiation. Analysis of mesodermal and neural crest progenitor markers showed that BMP4 treatment promoted expression of mesodermal markers GATA4, T (Brachyury homolog), and MSGN1 at the expense of neural crest markers p75 (NTR), HNK1, and Sox10.

A pericyte-like functionality of EDK and iPDK cells was further established in our co-culture experiments, as seen by the direct interaction between EDK and iPDK cells and endothelial cells in 3D fibrin-based tissue constructs, and by the ability of these cells to promote the formation of the vascular network. These results are consistent with previous reports showing that pericytes contribute to endothelial cell proliferation and survival and promote endothelial sprouting [2], [3], [33]. Confocal immunofluorescent analysis of vascular networks further demonstrated the deposition of Type IV Collagen and Laminin 5 at the interface between endothelial cells and EDK and iPDK, indicating the formation of vascular basement membrane. These results are consistent with previous reports showing that, in mature vasculature, pericytes provide supporting functions, including stabilization of blood vessels, formation of permeability barrier and deposition of vascular basement membrane proteins [14], [34], [35]. These results suggest that pericyte-like phenotype of hESC- and iPSC-derived fibroblasts is a key to controlling their ability to promote angiogenesis and to provide supporting functions for maturing vasculature.

Taken together, these findings demonstrate that highly angiogenic cells can be generated with high efficiency and purity from pluripotent stem cells using our directed differentiation system. These cells display a pericyte-like phenotype and stimulate and support different stages of the angiogenic process. Considering the limtations associated with the isolation and use of mesenchymal progenitor cells and pericytes from existing sources, such as limited growth and cell heterogeneity, the isolation of hESC- and hiPSC-derived fibroblasts that support angiogenesis may inform new advances in regenerative therapies.

## Supporting Information

Table S1 Primer sequences used for RT-PCR.
(DOCX)

Table S2 Human angiogenesis array alignment and coordinates.
(DOCX)

Video S1 Confocal movie demonstrating 3D vascular network within fibrin matrix. 3D vascular networks formed within fibrin matrix following seeding of RFP-HUVEC cells with EDK at ratio 3∶1 at day 8 post-seeding.
(AVI)
