# Supplementary material for: Fibroblasts Derived from Human Pluripotent Stem Cells Activate Angiogenic Responses In Vitro and In Vivo
Source: PLoS One. 2013 Dec 30;8(12):e83755. doi: 10.1371/journal.pone.0083755 (PMC3875480; doi:10.1371/journal.pone.0083755)
Supplement: Table S1 — Primer sequences used for RT-PCR. (DOCX) [file pone.0083755.s001.docx]

### Table S1. Primer sequences used for RT-PCR

| **Gene** | **Forward primer** | **Reverse primer** |
| --- | --- | --- |
| **GAPDH** | TCGACAGTCAGCCGCATCTTCTTT | ACCAAATCCGTTGACCTT |
| **CD31** | CAACGAGAAAATGTCAGA | GGAGCCTTCCGTTCTAGAGT |
| **CD34** | TGAAGCCTAGCCTGTCACCT | CGCACAGCTGGAGGTCTTAT |
| **VEGFR2** | GGCCCAATAATCAGAGTGGCA | TGTCATTTCCGATCACTTTTGGA |
| **CD146** | CTCGACTCCACAGTCTGGGAC | AAGGCAACCTCAGCCATGTCG |
| **PDGFRβ** | GTGGTGATCTCAGCCATCCT | CCGACATAAGGGCTTGCTT |
| **NG2** | GCTTTGACCCTGACTATGTTGGC | TCCAGAGTAGAGTCGCAGCA |
| **SM-MHC** | CGCCAAGAGACTCGTCTGG | TCTTTCCCAACCGTGACCTTC |
| **Calponin** | CAGTCCACCCTCCTGGCTTTG | GATGTTCCGCCCTTCTCTTAG |
| **Caldesmon** | AGTATGTGGGAGAAAGGGAATG- | AGGTTTGGGAGCAGGTGA |
| **SM22α** | AGGAGCGGCTGGTGGAGTGGAT | CATGTCAGTCTTGATGACCCCATAGT |
| **ACTA2** | CATCTCCAGAGTCCAGCACA | ACTGGGACGACATGGAAAAG |
| **P75(NTR)** | GGCTTCGGACCCCCGGTCTC | TCGTCTACTCGAGCTGGCCAATG |
| **HNK1** | TGTGAGTGCTGGTAATGAGGAGCC | GCAGAGTCCAGGGCAGCACG |
| **Sox10** | ATACGACACTGTCCCGGCCCTAAA | TTCTCCTCTGTCCAGCCTGTTCTC |
| **MSGN1** | TCTTCTTCTCCCTGTCCAGC | GTGTGTGAGTTCCCCGATGT |
| **GATA4** | GGAGGCGAGATGGGACGGGT | TGGGGACCCCGTGGAGCTT |
| **T** | TATTGGCAACTTTGGCACACCA | GGCTTCACTAATAACTGGACGAATCAC |
