# Supplementary material for: Fibroblasts Derived from Human Pluripotent Stem Cells Activate Angiogenic Responses In Vitro and In Vivo
Source: PLoS One. 2013 Dec 30;8(12):e83755. doi: 10.1371/journal.pone.0083755 (PMC3875480; doi:10.1371/journal.pone.0083755)
Supplement: Table S2 — Human angiogenesis array alignment and coordinates. (DOCX) [file pone.0083755.s002.docx]

## Table S2. Human angiogenesis array alignment and coordinates.

|  | **1** | **2** | **3** | **4** | **5** | **6** | **7** | **8** | **9** | **10** | **11** | **12** | **13** | **14** | **15** | **16** | **17** | **18** | **19** | **20** | **21** | **22** | **23** | **24** |
| --- | --- | --- | --- | --- | --- | --- | --- | --- | --- | --- | --- | --- | --- | --- | --- | --- | --- | --- | --- | --- | --- | --- | --- | --- |
| **A** | o | o |  |  | o | o | o | o | o | o | o | o | o | o | o | o | o | o | o | o |  |  | o | o |
| **B** | o | o | o | o | o | o | o | o | o | o | o | o | o | o | o | o | o | o | o | o | o | o | o | o |
| **C** | o | o | o | o | o | o | o | o | o | o | o | o | o | o | o | o | o | o | o | o | o | o | o | o |
| **D** | o | o | o | o | o | o | o | o | o | o | o | o | o | o | o | o | o | o | o | o | o | o | o | o |
| **E** | o | o | o | o | o | o | o | o | o | o | o | o | o | o | o | o | o | o | o | o | o | o |  |  |
| **F** | o | o |  |  |  |  |  |  |  |  |  |  |  |  |  |  |  |  |  |  |  |  | o | o |

| **Coordinate** | **Target/Control** | **Coordinate** | **Target/Control** |
| --- | --- | --- | --- |
| **A1, A2** | Reference Spots (+cntr) | **C17, C18** | IL-8 (CXCL8) |
| **A5, A6** | Activin A | **C19, C20** | LAP (TGF-β1) |
| **A7, A8** | ADAMTS-1 | **C21, C22** | Leptin |
| **A9, A10** | Angiogenin (ANG) | **C23, C24** | MCP-1 (CCL2) |
| **A11, A12** | Angiopoietin-1 (Ang-1 ) | **D1, D2** | MIP-1α (CCL3) |
| **A13, A14** | Angiopoietin-2 (Ang-2) | **D3, D4** | MMP-8 |
| **A15, A16** | Angiostatin/Plasminogen | **D5, D6** | D5, D6 MMP-9 |
| **A17, A18** | Amphiregulin (AR) | **D7, D8** | NRG1-β1 (HRG1-β1) |
| **A19, A20** | Artemin | **D9, D10** | Pentraxin 3 (PTX3) |
| **A23, A24** | Reference Spots (+cntr) | **D11, D12** | PD-ECGF |
| **B1, B2** | Coagulation Factor III (TF) | **D13, D14** | PDGF-AA |
| **B3, B4** | CXCL16 | **D15, D16** | PDGF-AB/PDGF-BB |
| **B5, B6** | DPPIV (CD26) | **D17, D18** | Persephin |
| **B7, B8** | EGF | **D19, D20** | Platelet Factor 4 (PF4) |
| **B9, B10** | EG-VEGF PK1 | **D21, D22** | PlGF |
| **B11, B12** | Endoglin (CD105) | **D23, D24** | Prolactin |
| **B13, B14** | Endostatin/Collagen XVIII | **E1, E2** | Serpin B5 (Maspin) |
| **B15, B16** | Endothelin-1 (ET-1) | **E3, E4** | Serpin E1 (PAI-1) |
| **B17, B18** | FGF acidic (FGF-1) | **E5, E6** | Serpin F1 (PEDF) |
| **B19, B20** | FGF basic (FGF-2) | **E7, E8** | TIMP-1 |
| **B21, B22** | FGF-4 | **E9, E10** | TIMP-4 |
| **B23, B24** | FGF-7 (KGF) | **E11, E12** | Thrombospondin-1 TSP-1 |
| **C1, C2** | GDNF | **E13, E14** | Thrombospondin-2 TSP-2 |
| **C3, C4** | GM-CSF | **E15, E16** | uPA |
| **C5, C6** | HB-EGF | **E17, E18** | Vasohibin |
| **C7, C8** | HGF | **E19, E20** | VEGF |
| **C9, C10** | IGFBP-1 | **E21, E22** | VEGF-C |
| **C11, C12** | IGFBP-2 | **F1, F2** | Reference Spots (+cntr) |
| **C13, C14** | IGFBP-3 | **F23, F24** | Negative Control |
| **C15, C16** | IL-1β (IL-1F2) |  |  |
